# Supplementary figures and images for: Conformational dynamics and allosteric modulation of the SARS-CoV-2 spike
Source: eLife. 2022 Mar 24;11:e75433. doi: 10.7554/eLife.75433 (PMC8963877; doi:10.7554/eLife.75433)

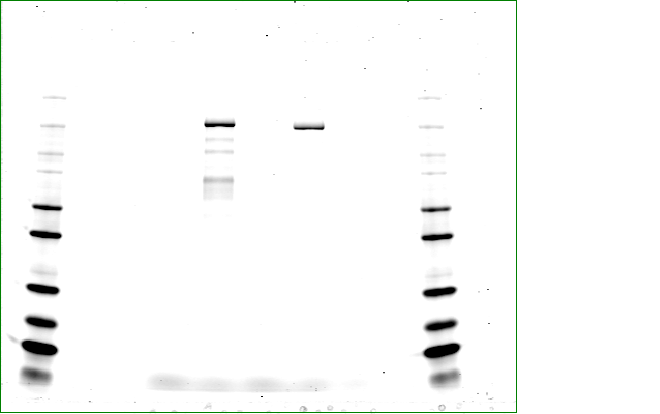

Supplement: Figure 1—figure supplement 1—source data 1. [file elife-75433-fig1-figsupp1-data1.zip › Figure 1- supplement 1 - source data S1G-3.bmp]

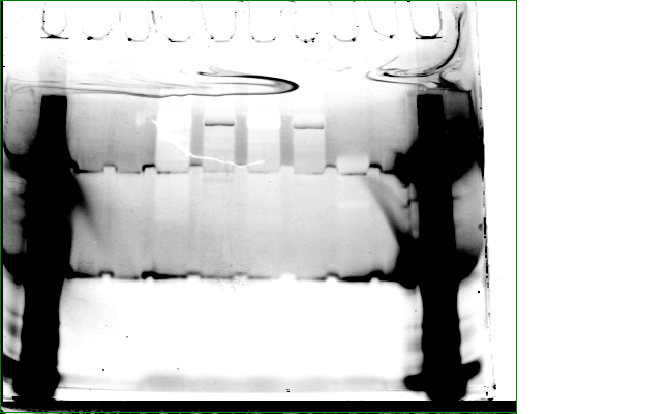

Supplement: Figure 1—figure supplement 1—source data 1. [file elife-75433-fig1-figsupp1-data1.zip › Figure 1- supplement 1 - source data S1G-2.bmp]

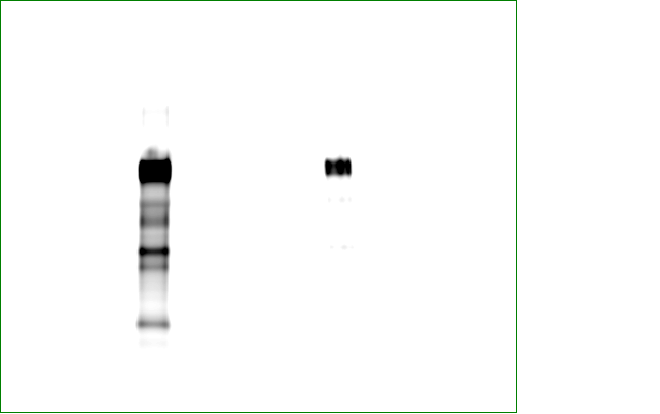

Supplement: Figure 1—figure supplement 1—source data 1. [file elife-75433-fig1-figsupp1-data1.zip › Figure 1- supplement 1 - source data S1D-2.bmp]
